# Associação entre Relação Glicose/Linfócito e Lesão Renal Aguda Induzida por Contraste em Pacientes com Infarto do Miocárdio Não Diabéticos

**DOI:** 10.36660/abc.20240869

**Published:** 2025-08-04

**Authors:** Murat Gök, Alparslan Kurtul, Orçun Demir, Kenan Yalta

**Affiliations:** 1 Trakya University Faculty of Medicine Edirne Turquia Trakya University Faculty of Medicine, Edirne – Turquia; 2 Mustafa Kemal Universitesi Tayfur Ata Sokmen Tip Fakultesi, Hatay Turquia Mustafa Kemal Universitesi Tayfur Ata Sokmen Tip Fakultesi, Hatay – Turquia

**Keywords:** Infarto do Miocárdio, Injúria Renal Aguda, Meios de Contraste

## Abstract

**Fundamento:**

O metabolismo da glicose e a inflamação sistêmica parecem estar fortemente relacionados a muitas doenças cardiovasculares. A relação glicose-linfócito (RGL), um novo marcador promissor, tem sido reconhecida como um preditor confiável de prognóstico em vários tipos de câncer. No entanto, ainda não existem estudos sobre a associação entre doenças cardiovasculares e GLR.

**Objetivos:**

Esta análise teve como objetivo investigar a possível associação entre RGL e o risco de lesão renal aguda induzida por contraste (LRAIC) após intervenção coronária percutânea primária (ICPP) em uma população de pacientes com infarto agudo do miocárdio com elevação do segmento ST (IAMST).

**Métodos:**

Os dados clínicos de 592 pacientes com IAMST não diabéticos tratados com ICPP entre fevereiro de 2021 e fevereiro de 2023 foram analisados retrospectivamente. Pacientes com doença renal terminal, dados laboratoriais ausentes, câncer, doenças inflamatórias/infecciosas ou que faleceram durante o procedimento ou dentro de 24 horas após o procedimento foram excluídos. A curva característica de operação do receptor (ROC) foi utilizada para determinar o valor de corte ideal da RGL na LRAIC. Com base nesse valor de corte, a população do estudo foi categorizada em grupos de RGL alta (≥4,16) e RGL baixa (<4,16). O nível de significância adotado na análise estatística foi de 5%.

**Resultados:**

A incidência geral de LRAIC foi de 7,4%. O grupo de RGL alta apresentou uma incidência maior de LRAIC em comparação ao grupo de RGL baixa (30,9% vs. 1,3%, p<0,001). Após ajuste para potenciais fatores de confusão, a RGL alta continuou sendo um preditor independente para LRAIC [razão de chances (OR) 45,100, intervalo de confiança (IC) 95% 7,312-278,174, p<0,001], assim como a creatinina na admissão (OR: 10,459, IC 95% 1,169-93,583, p=0,036).

**Conclusão:**

Em conclusão, a RGL elevada foi um fator de risco independente para o desenvolvimento de LRAIC ICPP em indivíduos com IAMST sem diabetes mellitus.

## Introdução

A lesão renal aguda induzida por contraste (LRAIC) é uma condição desafiadora, particularmente em pacientes com infarto agudo do miocárdio com elevação do segmento ST (IAMST), mesmo naqueles com função renal basal normal.^1^ A resposta inflamatória e o estresse oxidativo desempenham um papel significativo em sua fisiopatologia.^2 , 3^ O desenvolvimento de LRAIC tem sido fortemente associada a desfechos desfavoráveis, internação hospitalar prolongada e custos substanciais de saúde.^4 , 5^ Portanto, a detecção do risco de LRAIC e a implementação de algoritmos adequados de proteção renal podem melhorar substancialmente os desfechos clínicos em pacientes com IAMST.^6 - 8^ Assim, há uma necessidade evidente de biomarcadores novos e de fácil acesso para a rápida previsão do risco de LRAIC.

A relação glicose-linfócito (RGL) tem sido considerada um índice do metabolismo da glicose e da resposta inflamatória sistêmica, sendo relatada como um promissor índice prognóstico em pacientes com vários tipos de câncer.^9 , 10^ Além disso, dados recentes também sugerem que a RGL pode fornecer informações prognósticas importantes em pacientes criticamente enfermos com doenças inflamatórias agudas.^11 - 14^ Até o momento, existe apenas um estudo^15^ relatando o valor clínico da RGL pré-operatória na previsão da LRAIC em pacientes de unidade de terapia intensiva após cirurgia cardíaca. No entanto, não há nenhum estudo analisando a possível ligação entre RGL e LRAIC em pacientes com STEMI tratados com intervenção coronária percutânea primária (ICPP). Assim, neste estudo retrospectivo, analisamos o valor potencial da RGL na admissão para prever a ocorrência de LRAIC após ICPP em pacientes com IAMST sem diabetes mellitus, utilizando um banco de dados hospitalar retrospectivo.

## Métodos

### Seleção dos pacientes

**Figura 1:**
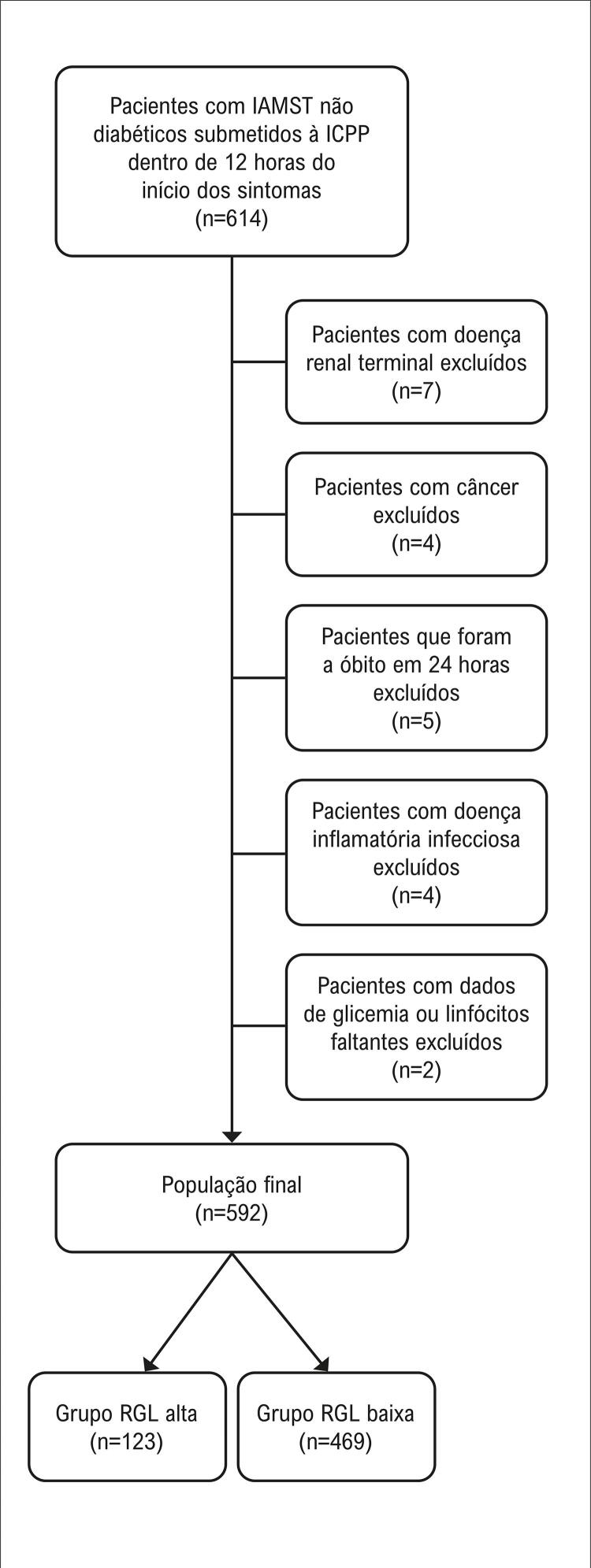
– Fluxograma da seleção dos pacientes; IAMST: infarto agudo do miocárdio com elevação do segmento ST; ICPP: intervenção coronária percutânea primária; RGL: relação glicose/linfócitos.

Um total de 614 indivíduos não diabéticos com IAMST submetidos à ICPP foram incluídos neste estudo retrospectivo. Pacientes com doença renal em estágio terminal (n=7), valores ausentes de glicose ou linfócitos (n=2), câncer (n=4), doença inflamatória/infecciosa (n=4) ou que faleceram durante o procedimento ou dentro de 24 horas após o procedimento (n=5) foram excluídos do estudo. Um total de 592 indivíduos foi incluído na análise final ( Figura 1 ). Este estudo foi aprovado pelo comitê de ética em pesquisa do Hospital Universitário Trakya, Turquia. Devido ao desenho retrospectivo, a exigência de um termo de consentimento informado foi dispensada.

Amostras de sangue venoso foram obtidas de todos os indivíduos antes da ICPP e 24, 48 e 72 horas após o procedimento para análise laboratorial. Dados demográficos, clínicos e laboratoriais foram obtidos dos prontuários médicos eletrônicos. Esses incluíram Índice de Massa Corporal (IMC), idade, gênero, hipertensão, tabagismo, níveis de colesterol total, lipoproteína de alta densidade, glicose sérica, lipoproteína de baixa densidade, creatinina, glóbulos brancos, linfócitos e de plaquetas, RGL, troponina T e proteína C reativa de alta sensibilidade. Um analisador hematológico automático e contador de células sanguíneas (XE-2100, Sysmex, Kobe, Japão) foi usado para análise dos parâmetros do hemograma completo. A RGL foi calculada usando glicose sanguínea na admissão (mmol/L)/contagem de linfócitos (× 10^9^/L).

O diagnóstico de IAMST foi baseado nos seguintes critérios sugeridos pela Sociedade Europeia de Cardiologia: (1) angina pectoris típica persistindo por mais de 30 minutos; (2) alterações dinâmicas no ECG (elevação do segmento ST no ponto J em pelo menos duas derivações contíguas, manifestando-se como uma elevação do segmento ST de 1,5 mm em mulheres, 2,5 mm em homens < 40 anos, 2 mm em homens ≥ 40 anos nas derivações V2–V3 e/ou 1 mm de elevação em outras derivações na ausência de bloqueio do ramo esquerdo); (3) elevação nos marcadores séricos de lesão miocárdica; (4) anatomia típica na artéria relacionada ao infarto, indicando intervenção coronária.^16^

A hipertensão foi definida como a média (de três medidas) da pressão arterial sistólica > 140 mmHg ou pressão arterial diastólica > 90 mmHg, ou o uso de medicação anti-hipertensiva. O diabetes mellitus foi definido como hemoglobina glicada ≥ 6,5%, glicemia de jejum ≥ 6,94 mmol/L, ou o uso de insulina ou outros medicamentos para diabetes. Pacientes já diagnosticados ou recém-diagnosticados com diabetes não foram incluídos no estudo. O tabagismo ativo foi definido como tabagismo regular nos últimos seis meses. O desfecho primário foi a evolução da LRAIC, definida principalmente como uma elevação absoluta da creatinina sérica ≥ 0,027 mmol/L ou uma elevação relativa da creatinina sérica ≥ 25% ocorrendo dentro de 48–72 horas após ICPP.^17^

Todos os pacientes receberam rotineiramente uma única dose de aspirina oral (300 mg), 600 mg de clopidogrel/180 mg de ticagrelor antes doa ICPP e 100 U/kg de heparina não fracionada intravenosa (doses adicionais foram administradas quando apropriado, para atingir um tempo de coagulação ativado > 250 segundos). A ICPP foi realizada via acesso transfemoral utilizando instrumentos padrões (fios-guia padrão, cateteres e *stents* farmacológicos). A decisão de usar antagonistas da glicoproteína IIb/IIIa foi deixada a critério do cardiologista operador. A hidratação intravenosa foi administrada conforme a preferência do cardiologista da unidade de terapia intensiva coronariana. O meio de contraste utilizado no procedimento foi não iônico e de baixa osmolaridade (Iohexol [Omnipaque; GE Healthcare Inc.]). O tempo total de isquemia (período desde o início dos sintomas até a reperfusão mecânica) também foi avaliado. A fração de ejeção do ventrículo esquerdo (FEVE) foi avaliada por ecocardiografia bidimensional.

### Análise estatística

O teste de Kolmogorov-Smirnov foi aplicado para verificar a distribuição normal das variáveis. As variáveis contínuas com distribuição normal foram expressas como média ± desvio padrão (DP) e comparadas usando o teste t para amostras independentes. As variáveis contínuas sem distribuição normal foram expressas como mediana (intervalo interquartil 25º-75º) e comparadas pelo teste U de Mann-Whitney. As variáveis categóricas foram expressas como contagens e porcentagens (%) e comparadas usando o teste do qui-quadrado. A curva característica de operação do receptor (ROC) foi utilizada para determinar o valor de corte ideal da RGL para LRAIC. Todos os fatores potenciais para LRAIC foram inicialmente avaliados por análise univariada. O nível de significância adotado na análise estatística foi de 5%. A regressão logística multivariada e a razão de chances (OR, *odds ratio* ) ajustada também foram realizadas na coorte do estudo para examinar a relação entre RGL e LRAIC. Todas as análises foram realizadas utilizando o pacote de software estatístico SPSS (SPSS Inc, Chicago, Illinois) versão 24.0, e um valor de P < 0,05 foi considerado estatisticamente significativo.

## Resultados

**Figura 2:**
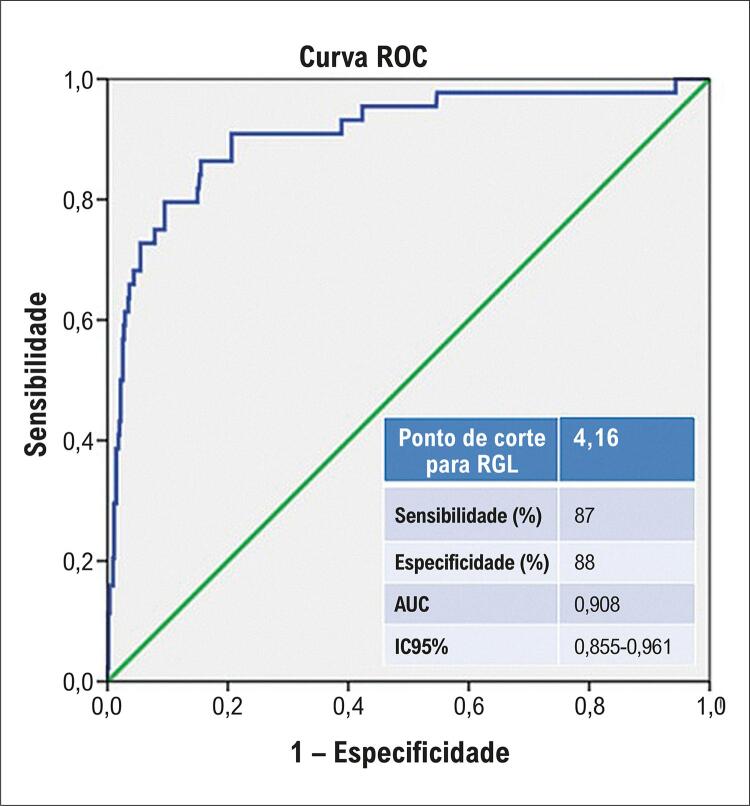
– Análise da curva ROC da Relação Glicose/Linfócito (RGL) na predição da lesão renal aguda induzida por contraste em pacientes com infarto agudo do miocárdio com elevação do segmento ST; AUC: área sob a curva; IC: intervalo de confiança.

Dos 592 indivíduos (idade média de 56,9 anos, 81,3% homens), a LRAIC foi observada em 44 (7,4%). As características basais estão listadas na Tabela 1 ; 63% (n=373) eram fumantes ativos e 30,9% (n=183) tinham hipertensão arterial. Na análise da curva ROC, o ponto de corte ideal para a RGL foi de 4,16, com boa sensibilidade (87%) e especificidade (88%) (AUC = 0,908, Figura 2 ). Os indivíduos foram categorizados em dois grupos com base no valor de corte da RGL – 123 pacientes no grupo “RGL alta” (≥4,16) e 469 pacientes no grupo “RGL baixa” (<4,16). O grupo com RGL alta apresentou uma incidência significativamente maior de LRAIC (p<0,001) ( Figura Central ). Os indivíduos do grupo RGL alta eram mais velhos e tinham uma prevalência significativamente menor de tabagismo ativo, além de uma maior porcentagem de mulheres, hipertensão arterial, lesões coronárias multivasculares e pré-dilatação com balão em comparação com o grupo com RGL baixa. Comparado ao grupo com RGL baixa, o grupo de indivíduos com RGL alta apresentou níveis significativamente mais elevados de glicose sérica, colesterol de lipoproteína de alta densidade, volume de contraste e tempo total de isquemia, além de menor FEVE, triglicerídeos, hemoglobina e contagem de linfócitos na admissão. A incidência de sobrevivência hospitalar também foi menor no grupo com RGL alta (p<0,001).

**Tabela 1:** – Comparações das características dos pacientes de acordo a relação glicose/linfócito (baixa ou alta)

Variáveis	Relação glicose/linfócito		Valor p
	Baixa (< 4,16) (n=469)	Alta (≥ 4,16) (n=123)	
Idade	54,8 ±11,8	64,7±13,2	<0,001
Mulheres	76 (16,2%)	35 (26,5%)	0,020
Tabagismo ativo	319(68,0%)	54 (43,9%)	<0,001
História familiar de doença arterial coronariana	166 (35,4%)	19 (15,4%)	<0,001
Hipertensão arterial	133(28,4%)	50 (40,7%)	0,007
Dislipidemia	110 (23,5%)	27 (22,0%)	0,413
Índice de massa corporal (Kg/m^2^)	27,8±3,8	27,1±4,3	0,152
Pressão arterial sistólica (mmHg)	127±22	126±25	0,635
Pressão arterial diastólica (mmHg)	77,8±13,5	76,4±15,4	0,380
Frequência cardíaca (batimentos/minuto)	77,6±14,7	80,1±16,5	0,152
Fração de ejeção ventricular esquerda (%)	47,8±9,2	43,7±10,8	<0,001
Colesterol total (mmol/L)	10,50 ± 2,33	10,33 ± 2,39	0,478
Lipoproteína de baixa densidade (mmol/L)	3,13 ± 0,93	3,08 ± 0,96	0,578
Lipoproteína de alta densidade (mmol/L)	0,98 ± 0,21	1,06 ± 0,21	0,002
Triglicerídeos (mmol/L)	3,78(2,66-5,12)	3,0 (2,09-4,47)	0,001
Hemoglobina (g/L)	0,147 ±0,015	0,137 ± 0,022	<0,001
Contagem de leucócitos (x10^9^/L)	11,8 ±3,6	10,9 ± 4,1	0,237
Contagem de linfócitos (x10^9^/L)	2,8 (2,2-3,8)	1,4 (0,9-1,8)	<0,001
Contagem de plaquetas (x10^9^/L)	245 (211-291)	233 (187-279)	0,109
Glicemia na admissão (mmol/L)	6,28 (5,50-7,28)	7,78 (6,33-9,78)	<0,001
Relação glicose/linfócito na admissão	40 (30-53)	100 (83-143)	<0,001
Creatinina sérica na admissão (µmol/L)	90,1 ± 16,7	97,2 ± 26,5	0,012
Proteína C reativa ultrassensível (mg/L)	6,02 (2,4-10,6)	6,9 (3,5-10,6)	0,272
Pico de troponina T (ng/L)	2,813 (6,35-9,702)	4,767 (1,159-10,000)	0,120
Uso de IECA / BRA	387 (82,5%)	90 (73,2%)	0,016
Uso de estatina	443 (94,5%)	116 (94,3%)	0,547
Lesões coronárias em múltiplos vasos	210 (44,8%)	73 (59,3%)	0,003
Patência da artéria relacionada ao infarto	169 (36,0%)	32 (26,0%)	0,220
Pré-dilatação com balão	255 (54,4%)	82 (66,7%)	0,009
Volume de meio de contraste (mL)	140 (100-200)	150 (110-200)	0,001
Tempo total de isquemia (min)	120 (90-180)	120 (90-240)	0,066
Diâmetro do *stent* (mm)	3,17 ± 0,46	3,15 ± 0,47	0,722
Comprimento do *stent* (mm)	26,8 ± 12,3	27,3 ± 14,1	0,693
**Artéria culpada**			
Artéria descendente anterior esquerda	207 (44,1%)	50 (40,7%)	0,458
Artéria circunflexa esquerda	96 (20,5%)	22 (17,9%)	
Artéria direita	166 (35,4%)	51 (41,5%)	
LRAIC	6 (1,3%)	38(30,9%)	<0,001
Sobrevida no hospital	458 (97,7%)	106 (86,2%)	<0,001

BRA: bloqueador do receptor de angiotensina; IECA: inibidor de enzima conversora de angiotensina; LRAIC: lesão renal aguda induzida por contraste; valores em média ± DP, mediana (intervalo interquartil ou número (%).

**Figura Central:**
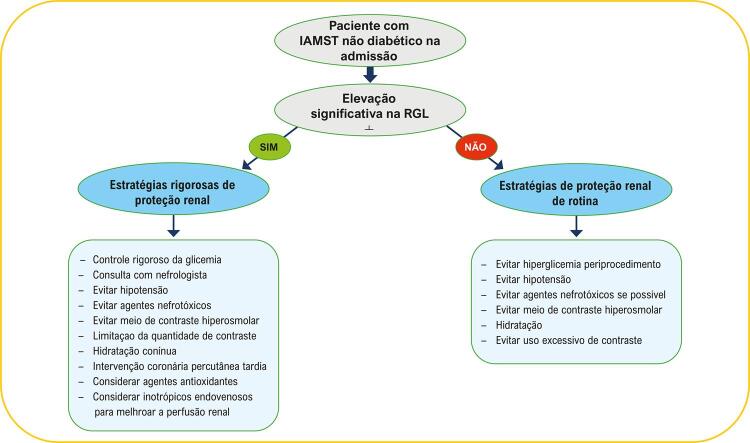
Associação entre Relação Glicose/Linfócito e Lesão Renal Aguda Induzida por Contraste em Pacientes com Infarto do Miocárdio Não Diabéticos

A análise de regressão logística univariada revelou que a RGL elevada, idade, gênero feminino, alto IMC, hipertensão arterial, tabagismo ativo, contagem de plaquetas, hemoglobina, creatinina sérica, FEVE e volume de meio de contraste estavam relacionados com o aumento da incidência de LRAIC. Esse achado foi corroborado pelos resultados da análise multivariada. A RGL elevada continuou sendo um preditor independente de LRAIC na coorte do estudo após o ajuste, com um OR de 45,100 (p<0,001), juntamente com a creatinina (OR: 10,459, p=0,036) após ajuste para IMC, gênero, idade, comorbidades, FEVE, quantidade total de contraste e resultados laboratoriais ( Tabela 2 ).

**Tabela 2:** – Análise de regressão univariada e multivariada para lesão renal aguda induzida por contraste em pacientes com infarto agudo do miocárdio com elevação do segmento ST

Variável	Análise univariada		Análise multivariada	
	Odds ratio (IC95%)	p	Odds ratio (IC95%)	p
Idade	1,095 (1,068-1,123)	<0,001	1,053 (0,977-1,135)	0,180
Mulheres	3,387 (1,784-6,430)	<0,001	2,233 (0,318-15,685)	0,419
Índice de massa corporal	0,904 (0,818-0,998)	0,046	0,905 (0,738-1,109)	0,336
Hipertensão arterial	3,597 (1,918-6,747)	<0,001	2,205 (0,437-11,131)	0,338
Hiperlipidemia	0,503 (0,208-1,216)	0,127		
Tabagismo ativo	0,219 (0,112-0,428)	<0,001	0,562 (0,67-4,6866)	0,594
Leucócitos	1,064 (0,992-1,142)	0,083		
RGL ≥ 4,16	34,498 (14,145-84,136)	<0,001	45,100 (7,312-278,174)	<0,001
Contagem de plaquetas	0,994 (0,989-0,999)	0,019	0,992 (0,980-1,003)	0,159
PCR-us	1,008 (0,987-1,029)	0,469		
Creatinina sérica	30,623 (9,659-97,090)	<0,001	10,459 (1,169-93,583)	0,036
Hemoglobina	0,967 (0,572-0,777)	<0,001	0,877 (0,570-1,350)	0,551
Pressão arterial sistólica	0,985 (0,971-1,001)	0,060		
Pressão arterial diastólica	0,978 (0,955-1,001)	0,065		
Frequência cardíaca	1,009 (0,989-1,030)	0,376		
FEVE	0,924 (0,893-0,956)	<0,001	0,921 (0,848-1,001)	0,053
Tempo total de isquemia	1,002 (0,999-1,004)	0,170		
Volume de meio de contraste	1,008 (1,004-1,012)	<0,001	1,007 (0,998-1,015)	0,116
Lesões coronárias em múltiplos vasos	0,611 (0,328-1,141)	0,122		
Diâmetro do stent	0,714 (0,340-1,499)	0,374		
Comprimento do stent	1,005 (0,979-1,030)	0,722		

IC: intervalo de confiança; RGL: relação glicose/linfócito; PCR-us: Proteína C-reativa Ultrassensível; FEVE: fração de ejeção ventricular esquerdo.

## Discussão

A presente análise sugere que a RGL é um preditor independente de LRAIC em pacientes com IAMST não diabéticos tratados com ICPP. Esse achado pode ter implicações diagnósticas, terapêuticas e prognósticas importantes nesse grupo de pacientes.

A incidência de LRAIC após ICPP depende amplamente das características clínicas e demográficas basais, juntamente com fatores que surgem durante a intervenção.^8^ Em concordância com estudos anteriores, a incidência de LRAIC foi de 7,4% em nosso estudo. Os mecanismos da LRAIC incluem principalmente a toxicidade tubular renal direta exercida pelo contraste e alterações hemodinâmicas renais que levam à hipóxia medular e à inflamação,^18^ e posteriormente à liberação de radicais de oxigênio, e de substâncias vasoconstritoras e trombogênicas.^19^

A LRAIC tem sido um fator de risco para desfechos desfavoráveis, independentemente da função renal basal, e uma limitação potencial para intervenções cardiovasculares invasivas, especialmente em pacientes com IAMST.^1 , 2 , 20^ A mitigação do desenvolvimento de LRAIC em pacientes com IAMST pode reduzir significativamente eventos adversos pós-procedimento e, assim, melhorar os desfechos clínicos. Muitos pesquisadores demonstraram que certos biomarcadores inflamatórios derivados do hemograma podem desempenhar um papel crucial na previsão do risco de LRAIC no contexto do IAMST.^21 - 23^ No entanto, apenas alguns estudos analisaram o impacto combinado de fatores inflamatórios e glicose nesse contexto.

A RGL é um índice inovador que reflete tanto o metabolismo da glicose quanto a resposta inflamatória sistêmica e tem sido sugerido como um marcador de risco promissor no contexto de malignidade e unidade de terapia intensiva.^9 - 14^ No entanto, poucos estudos exploraram a relevância da RGL nas doenças cardiovasculares. Importante destacar que este parece ser o primeiro estudo a explorar a relação entre a RGL pré-intervencional e o desenvolvimento de LRAIC em pacientes com IAMST (submetidos à ICPPI), sugerindo que a RGL pode ser um preditor independente de LRAIC nesses indivíduos de alto risco. Portanto, este estudo aumentou ainda mais a utilidade clínica desse índice.

Os mecanismos absolutos sobre a relação independente entre RGL e LRAIC ainda são desconhecidos. Por outro lado, a glicose sérica é considerada um marcador metabólico potencialmente associado à indução de uma inflamação sutil e persistente.^24^ Evidências crescentes demonstraram que níveis elevados de glicose podem desencadear estresse oxidativo e inflamação crônica associada, que pode se manifestar pela expressão de vários mediadores pró-inflamatórios.^25 , 26^ Em circunstâncias de hiperglicemia, células endoteliais ativadas podem liberar citocinas pró-inflamatórias em resposta a sinais parácrinos e autócrinos, geralmente demonstrando um ciclo vicioso.^27^ Além disso, a hiperglicemia pode induzir a oxidação da glicose juntamente com a glicação não enzimática de proteínas, levando a uma produção desproporcionalmente alta de radicais livres.^28^ No entanto, mecanismos de defesa antioxidantes podem ter o potencial de prevenir tais danos patológicos e a resistência emergente à insulina, ainda mais agravada pelo estresse oxidativo associado à hiperglicemia.^29^

Por outro lado, como um componente crucial da resposta inflamatória sistêmica, a diminuição da contagem de linfócitos pode estar associada a um prognóstico cardiovascular ruim no IAMST.^30^ Diversos estudos sugeriram biomarcadores inflamatórios séricos baseados em linfócitos (incluindo a razão neutrófilo-linfócito,^21^ o índice de inflamação imunológica sistêmica,^22^ o índice nutricional prognóstico,^23^ entre outros) como marcadores do desenvolvimento de LRAIC e desfechos desfavoráveis em pacientes de diversas populações. No entanto, apenas alguns estudos investigaram o impacto combinado de fatores pró-inflamatórios e glicose como fator metabólico. Em conjunto, a RGL representa o impacto sinérgico da inflamação sistêmica e da glicose, sugerindo seu valor clínico único. No entanto, o valor clínico da RGL na previsão de LRAIC deve ser mais amplamente testado em pacientes com IAMST, bem como em outras populações.

Este estudo também apresenta algumas limitações. Primeiro, trata-se de uma análise de um único centro, o que pode estar inerentemente associado a vieses de seleção, mesmo com um grande tamanho amostral. Segundo, não foi possível avaliar as mudanças seriadas no valor da RGL durante a internação hospitalar. Terceiro, os níveis de creatinina sérica podem ser influenciados por alterações hemodinâmicas, embora nenhum paciente da coorte do estudo tenha apresentado choque cardiogênico. Por fim, trata-se de uma análise retrospectiva, o que pode justificar a realização de estudos multicêntricos prospectivos para confirmar essas descobertas.

## Conclusões

No presente estudo, mostramos, pela primeira vez, que a RGL na admissão pode atuar como um preditor independente de LRAIC pós-procedimento em indivíduos com IAMST tratados com ICPP. Esses resultados podem impactar a prática clínica, incluindo a possibilidade de utilizar a RGL em protocolos clínicos e estratégias de proteção renal, como hidratação intravenosa e uso limitado de meio de contraste durante o procedimento. Estudos prospectivos adicionais são necessários para entender melhor o mecanismo da relação entre RGL elevada e LRAIC.

## References

[1] Khalfallah M, Abdalaal M, Adel M (2019). Contrast-Induced Nephropathy in Patients with ST-Segment Elevation Myocardial Infarction: Is it Affected by Treatment Strategy?. Glob Heart.

[2] Marenzi G, Cosentino N, Bartorelli AL (2015). Acute Kidney Injury in Patients with Acute Coronary Syndromes. Heart.

[3] La Manna G, Pancaldi LG, Capecchi A, Maska E, Comai G, Cappuccilli ML (2010). Risk for Contrast Nephropathy in Patients Undergoing Coronarography. Artif Organs.

[4] Rear R, Bell RM, Hausenloy DJ (2016). Contrast-Induced Nephropathy Following Angiography and Cardiac Interventions. Heart.

[5] Hobson C, Ozrazgat-Baslanti T, Kuxhausen A, Thottakkara P, Efron PA, Moore FA (2015). Cost and Mortality Associated with Postoperative Acute Kidney Injury. Ann Surg.

[6] Abe D, Sato A, Hoshi T, Kakefuda Y, Watabe H, Ojima E (2014). Clinical Predictors of Contrast-Induced Acute Kidney Injury in Patients Undergoing Emergency versus Elective Percutaneous Coronary Intervention. Circ J.

[7] Collister D, Pannu N, Ye F, James M, Hemmelgarn B, Chui B (2017). Health Care Costs Associated with AKI. Clin J Am Soc Nephrol.

[8] Khalfallah M, Allaithy A, Maria DA (2021). Incidence, Predictors and Outcomes of Contrast Induced Nephropathy in Patients with ST Elevation Myocardial Infarction Undergoing Primary Percutaneous Coronary Intervention. Glob Heart.

[9] Zhong A, Cheng CS, Kai J, Lu R, Guo L (2020). Clinical Significance of Glucose to Lymphocyte Ratio (GLR) as a Prognostic Marker for Patients with Pancreatic Cancer. Front Oncol.

[10] Navarro J, Kang I, Hwang HK, Yoon DS, Lee WJ, Kang CM (2019). Glucose to Lymphocyte Ratio as a Prognostic Marker in Patients with Resected pT2 Gallbladder Cancer. J Surg Res.

[11] Li L, Zou G, Liu J (2021). Preoperative Glucose-to-Lymphocyte Ratio is an Independent Predictor for Acute Kidney Injury after Cardiac Surgery in Patients in Intensive Care Unit. Int J Gen Med.

[12] Cai S, Wang Q, Ma C, Chen J, Wei Y, Zhang L (2022). Association between Glucose-to-Lymphocyte Ratio and in-Hospital Mortality in Intensive Care Patients with Sepsis: A Retrospective Observational Study Based on Medical Information Mart for Intensive Care IV. Front Med.

[13] Hu T, Liu X, Liu Y (2022). Usefulness of Glucose to Lymphocyte Ratio to Predict in-Hospital Mortality in Patients with AECOPD Admitted to the Intensive Care Unit. COPD.

[14] Zhang Y, Zhang S (2022). Prognostic Value of Glucose-to-Lymphocyte Ratio in Critically Ill Patients with Acute Respiratory Distress Syndrome: A Retrospective Cohort Study. J Clin Lab Anal.

[15] Yun KH (2018). Contrast-Induced Acute Kidney Injury and Inflammation. Korean Circ J.

[16] Ibánez B, James S, Agewall S, Antunes MJ, Bucciarelli-Ducci C, Bueno H (2017). 2017 ESC Guidelines for the Management of Acute Myocardial Infarction in Patients Presenting with ST-Segment Elevation. Rev Esp Cardiol.

[17] Stacul F, van der Molen AJ, Reimer P, Webb JA, Thomsen HS, Morcos SK (2011). Contrast Induced Nephropathy: Updated ESUR Contrast Media Safety Committee guidelines. Eur Radiol.

[18] Bansal S, Patel RN (2020). Pathophysiology of Contrast-Induced Acute Kidney Injury. Interv Cardiol Clin.

[19] Akcay A, Nguyen Q, Edelstein CL (2009). Mediators of Inflammation in Acute Kidney Injury. Mediators Inflamm.

[20] Sato A, Aonuma K, Watanabe M, Hirayama A, Tamaki N, Tsutsui H (2017). Association of Contrast-Induced Nephropathy with Risk of Adverse Clinical Outcomes in Patients with Cardiac Catheterization: From the CINC-J Study. Int J Cardiol.

[21] Tanik VO, Çinar T, Velibey Y, Öz A, Kalenderoglu K, Gümüsdag A (2019). Neutrophil-to-Lymphocyte Ratio Predicts Contrast-Induced Acute Kidney Injury in Patients with ST-Elevation Myocardial Infarction Treated with Primary Percutaneous Coronary Intervention. J Tehran Heart Cent.

[22] Karauzum I, Karauzum K, Hanci K, Gokcek D, Kalas B, Ural E (2022). The Utility of Systemic Immune-Inflammation Index for Predicting Contrast-Induced Nephropathy in Patients with ST-Segment Elevation Myocardial Infarction Undergoing Primary Percutaneous Coronary Intervention. Cardiorenal Med.

[23] Kurtul A, Gok M, Esenboga K (2021). Prognostic Nutritional Index Predicts Contrast-Associated Acute Kidney Injury in Patients with ST-Segment Elevation Myocardial Infarction. Acta Cardiol Sin.

[24] Hotamisligil GS (2017). Inflammation, Metaflammation and Immunometabolic Disorders. Nature.

[25] Zhao M, Wang S, Zuo A, Zhang J, Wen W, Jiang W (2021). HIF-1a/JMJD1A Signaling Regulates Inflammation and Oxidative Stress Following Hyperglycemia and Hypoxia-Induced Vascular Cell Injury. Cell Mol Biol Lett.

[26] Escobar-Morreale HF, Martínez-García MÁ, Montes-Nieto R, Fernández-Durán E, Temprano-Carazo S, Luque-Ramírez M (2017). Effects of Glucose Ingestion on Circulating Inflammatory Mediators: Influence of Sex and Weight Excess. Clin Nutr.

[27] Wang Y, Li J, Huang Y, Dai X, Liu Y, Liu Z (2017). Tripartite Motif-Containing 28 Bridges Endothelial Inflammation and Angiogenic Activity by Retaining Expression of TNFR-1 and -2 and VEGFR2 in Endothelial Cells. FASEB J.

[28] Khan MI, Rath S, Adhami VM, Mukhtar H (2018). Hypoxia Driven Glycation: Mechanisms and Therapeutic Opportunities. Semin Cancer Biol.

[29] Luna C, Estévez M (2018). Oxidative Damage to Food and Human Serum Proteins: Radical-Mediated Oxidation vs. Glyco-Oxidation. Food Chem.

[30] Núñez J, Núñez E, Bodí V, Sanchis J, Mainar L, Miñana G (2010). Low Lymphocyte Count in Acute Phase of ST-Segment Elevation Myocardial Infarction Predicts Long-Term Recurrent Myocardial Infarction. Coron Artery Dis.

