# Supplementary material for: Associação entre Relação Glicose/Linfócito e Lesão Renal Aguda Induzida por Contraste em Pacientes com Infarto do Miocárdio Não Diabéticos
Source: Arq Bras Cardiol. 2025 Aug 4;122(8):e20240869. [Article in Portuguese] doi: 10.36660/abc.20240869 (PMC12677807; doi:10.36660/abc.20240869)
Supplement: Translation

# Translation: Association between Glucose/lymphocytes Ratio and Contrast-Induced Acute Kidney Injury in Patients with Myocardial Infarction without Diabetes Mellitus

**DOI:** 10.36660/abc.20240869i

**Authors:** Murat Gök, Alparslan Kurtul, Orçun Demir, Kenan Yalta

**Affiliations:** 1 Türkiye Trakya University Faculty of Medicine, Edirne – Türkiye; 2 Türkiye Mustafa Kemal Universitesi Tayfur Ata Sokmen Tip Fakultesi, Hatay – Türkiye

**Keywords:** Myocardial Infarction, Acute Kidney Injury, Contrast Media

## Abstract

**Background:**

Glucose metabolism and systemic inflammation appears to be strongly related to many cardiovascular diseases. Glucose to lymphocyte ratio (GLR), a novel promising marker, has been recognized as a reliable predictor of prognosis in various cancers. However, there are still no studies on the association of cardiovascular disease with GLR.

**Objectives:**

This analysis aimed to uncover the potential association between GLR and the risk for contrast-induced acute kidney injury (CI-AKI) after primary percutaneous coronary intervention (PPCI) in a ST-elevation acute myocardial infarction (STEMI) population.

**Methods:**

Clinical data of 592 nondiabetic STEMI patients managed with PPCI from February 2021 to February 2023 were retrospectively analyzed. Patients with end-stage kidney disease, missing laboratory data, cancers, inflammatory/infectious diseases, or died during the procedure or within 24 hours after the procedure were excluded. The receiver operating characteristic curve was used to determine the optimal cutoff of GLR in CI-AKI. Based on the cutoff value, the study population was categorized into high-GLR (≥4.16) and low-GLR (<4.16) groups. The level of significance adopted in the statistical analysis was 5%.

**Results:**

The overall CI-AKI incidence was 7.4%. The high-GLR group showed a higher CI-AKI incidence in comparison to the low-GLR group (30.9%vs1.3%, p<0.001). Following adjustment for potential confounders, high-GLR still served as an independent predictor for CI-AKI (odds ratio [OR] 45.100, 95% confidence interval [CI] 7.312-278.174, p<0.001), as well as creatinine at admission (OR:10.459, 95%CI 1.169-93.583, p=0.036).

**Conclusions:**

In conclusion, a high GLR level served as an independent risk factor for CI-AKI evolution after PPCI in subjects with STEMI without diabetes mellitus.

## Introduction

Contrast-induced acute kidney injury (CI-AKI) has been a challenging condition particularly in subjects with ST segment elevation myocardial infarction (STEMI) even in those with normal renal functions at baseline.^1^Inflammatory response and oxidative stress play a significant role in its pathophysiology.^2 , 3^CI-AKI has been strongly associated with unfavorable outcomes, prolonged hospital stay as well as substantial healthcare costs.^4 , 5^Therefore, timely detection of CI-AKI risk and implementation of proper reno-protective algorithms may substantially improve clinical outcomes in patients with STEMI.^6 - 8^Accordingly, there exists an obvious need for novel and easily accessible biomarkers for the quick prediction of CI-AKI risk in this context.

The glucose-to-lymphocyte ratio (GLR) has been regarded as an index of glucose metabolism and systemic inflammatory response, and has been reported to serve as a promising prognostic index in subjects with various cancer types.^9 , 10^Furthermore, recent data also suggest that GLR could provide important prognostic information in critically ill patients with acute inflammatory diseases.^11 - 14^To date, there is only one study^15^reporting the clinical value of preoperative GLR in predicting postoperative CI-AKI in intensive care unit patients following cardiac surgery. However, there has been no single study analyzing the potential link between GLR and CI-AKI in STEMI patients managed with primary percutaneous coronary intervention (PPCI). Accordingly, in this retrospective study, we analyzed the potential value of admission GLR in predicting CI-AKI following PPCI in patients with STEMI without diabetes mellitus using a retrospective hospital database.

## Methods

### Selection of Patients

A total of 614 nondiabetic subjects with STEMI undergoing PPCI were included in this retrospective study. Patients with end-stage kidney disease (n=7), missing values of glucose or lymphocyte (n=2), with known cancer (n=4), inflammatory/infectious disease (n=4), or died during the procedure or within 24 hours after the procedure (n=5) were excluded from the study. A total of 592 subjects were included in the final analysis ( Figure 1 ). This study was approved by the research ethics committee of the Trakya University Hospital, Turkey. Due to the retrospective design, the requirement to sign an informed consent form was waived.

**Figure 1:** – Study flow diagram; STEMI: ST-Elevation myocardial infarction, PPCI: Primary percutaneous coronary intervention, GLR: glucose-to-lymphocyte ratio.

Venous blood samples were obtained from all subjects before PPCI and at 24, 48 and 72 h following the procedure for laboratory analysis. Demographic data, medical features and laboratory data were obtained from electronic medical records. These included: body mass index (BMI), age, gender, hypertension, smoking status, high-density lipoprotein-cholesterol, total cholesterol, serum glucose, low-density lipoprotein-cholesterol, creatinine, white blood cell, lymphocyte, and platelet levels, GLR, troponin t and high- sensitive C reactive protein. An automatic hematology analyzer and blood cell counter (XE-2100, Sysmex, Kobe, Japan) was used for the analysis of complete blood cell parameters. The GLR was calculated using admission blood glucose (mmol/L)/lymphocyte count (× 10^9^/L).

STEMI diagnosis was based on the following criteria suggested by the European Society of Cardiology: (1) typical angina pectoris persisting more than 30 min.; (2) dynamic alterations on ECG (ST-segment elevation at the J-point at least in two contiguous leads manifesting as an ST-segment elevation of 1.5 mm in women, 2.5 mm in men < 40 years, 2 mm in men 40 years in the leads V2–V3 and/or 1 mm elevation in other leads in those without left bundle branch block); (3) elevation in serum markers of myocardial injury; (4) typical anatomy in the infarct-related artery indicating coronary intervention.^16^

Hypertension was defined as the average (of three measurements) systolic blood pressure > 140 mmHg or diastolic blood pressure > 90 mmHg or the use of antihypertensive medication. Diabetes mellitus was defined as glycohemoglobin ≥ 6.5%, fasting blood glucose ≥ 6.94 mmol/L, or the use of insulin or other diabetes medication. Patients already diagnosed with or newly diagnosed with diabetes were not included in the study. Active smoking was defined as regular smoking within the past six months. The primary outcome was CI-AKI evolution primarily defined as an absolute serum creatinine elevation of ≥ 0.027 mmol/L or a relative elevation in serum creatinine ≥ 25% within 48–72 h following the PPCI.^17^

All patients were routinely administered a single dose of oral aspirin (300 mg), 600 mg of clopidogrel/180 mg of ticagrelor preceding the PPCI, and 100 U/kg of intravenous unfractioned heparin (additional doses were administered, where appropriate, to attain an activated clotting time value of > 250 seconds). PPCI was implemented via the transfemoral route using standard clinical tools (standard guidewires, catheters and drug-eluting stents). The decision to use glycoprotein IIb/IIIa antagonists was left to the discretion of the operating cardiologist. Intravenous hydration was given following the coronary intensive care unit cardiologist’s preference. The contrast medium used in the procedure was non-ionic and low-osmolar (Iohexol [Omnipaque; GE Healthcare Inc.]) The total ischemic time (the period starting from symptom onset to mechanical reperfusion) was also evaluated. The left ventricular ejection fraction (LVEF) was evaluated with 2-dimensional echocardiography.

### Statistical analysis

The Kolmogorov-Smirnov test was applied to verify the normal distribution of the variables. Continuous variables having normal distribution were expressed as mean ± standard deviation (SD) and were compared using the independent sample t-test. Continuous variables in the absence of normal distribution were expressed as median (25th-75th interquartile range) and were compared by the Mann–Whitney U-test. Categorical variables were expressed as counts and percentages (%) and were compared using the chi-square test. The receiver operating characteristic (ROC) curve was used to determine the optimal cutoff value of GLR for CI-AKI. All potential factors for CI-AKI were first evaluated by univariate analysis. The level of significance adopted in the statistical analysis was 5%. Multivariate logistic regression and adjusted odds ratio (OR) were also performed in the study cohort to examine the relationship between GLR and CI-AKI. All analyses were performed using the SPSS (SPSS Inc, Chicago, Illinois) 24.0 statistical software package, and a p<0.05 was considered statistically significant.

## Results

Of the 592 subjects (mean age 56.9 years, 81.3% men), CI-AKI was observed in 44 (7.4%). The baseline characteristics were listed in Table 1 ; 63% (n=373) were active smokers and 30.9% (n=183) had arterial hypertension. In ROC curve analysis, the optimal cutoff value for the GLR was found to be 4.16, with good sensitivity (87%) and specificity (88%) (AUC = 0.908, Figure 2 ). The subjects were categorized into two groups based on the optimal cutoff of GLR – 123 patients in the high-GLR group (≥4.16), and 469 patients in the low-GLR group (<4.16). High-GLR group was found to have a significantly higher incidence of CI-AKI (p<0.001) ( Central Illustration ). The subjects in the high GLR group were older and had a significantly lower prevalence of active smoking and a higher percentage of women, arterial hypertension, multivessel coronary lesions and balloon predilatation compared with low GLR group. Compared with the low-GLR group, the high-GLR group was found to have significantly higher serum glucose, high-density lipoprotein cholesterol, contrast media volume and total ischemia time, and lower LVEF, triglyceride, lower hemoglobin, lymphocyte count at admission. The incidence of in-hospital survival was also lower in the high-GLR group (p<0.001).

**Table 1:** – Comparisons of patient’s characteristics based on glucose to lymphocyte ratio cutoff

Variables	Glucose to lymphocyte ratio		Valor p
	Low (< 4.16) (n=469)	High (≥ 4.16) (n=123)	
Age	54.8 ±11.8	64.7±13.2	<0.001
Women	76 (16.2%)	35 (26.5%)	0.020
Active smoker	319(68.0%)	54 (43.9%)	<0.001
Family history of coronary artery disease	166 (35.4%)	19 (15.4%)	<0.001
Arterial hypertension	133(28.4%)	50 (40.7%)	0.007
Dyslipidemia	110 (23.5%)	27 (22.0%)	0.413
Body mass index (kg/m^2^)	27.8±3.8	27.1±4.3	0.152
Systolic blood pressure (mmHg)	127±22	126±25	0.635
Diastolic blood pressure (mmHg)	77.8±13.5	76.4±15.4	0.380
Pulse rate (beat/min)	77.6±14.7	80.1±16.5	0.152
Left ventricular ejection fraction (%)	47.8±9.2	43.7±10.8	<0.001
Total cholesterol (mmol/L)	10.50 ± 2.33	10.33 ± 2.39	0.478
Low-density lipoprotein cholesterol (mmol/L)	3.13 ± 0.93	3.08 ± 0.96	0.578
High-density lipoprotein cholesterol (mmol/L)	0.98 ± 0.21	1.06 ± 0.21	0.002
Triglyceride (mmol/L)	3.78(2.66-5.12)	3.0 (2.09-4.47)	0.001
Hemoglobin (g/L)	0.147 ±0.015	0.137 ± 0.022	<0.001
White blood cell count (x10^9^/L)	11.8 ±3.6	10.9 ± 4.1	0.237
Lymphocyte count (x10^9^/L)	2.8 (2.2-3.8)	1.4 (0.9-1.8)	<0.001
Platelet count (x10^9^/L)	245 (211-291)	233 (187-279)	0.109
Serum glucose at admission (mmol/L)	6.28 (5.50-7.28)	7.78 (6.33-9.78)	<0.001
Glucose to lymphocyte ratio	40 (30-53)	100 (83-143)	<0.001
Serum creatinine at admission (µmol/L)	90.1 ± 16.7	97.2 ± 26.5	0.012
High sensitive C-reactive protein (mg/L)	6.02 (2.4-10.6)	6.9 (3.5-10.6)	0.272
Peak troponin T value (ng/L)	2.813 (6.35-9.702)	4.767 (1.159-10.000)	0.120
ACEI / ARB use	387 (82.5%)	90 (73.2%)	0.016
Statin use	443 (94.5%)	116 (94.3%)	0.547
Multivessel coronary lesions	210 (44.8%)	73 (59.3%)	0.003
Initial patency of infarct-related artery	169 (36.0%)	32 (26.0%)	0.220
Balloon predilatation	255 (54.4%)	82 (66.7%)	0.009
Contrast media volume (ml)	140 (100-200)	150 (110-200)	0.001
Total ischemia time (min)		120 (90-240)	0.066
Stent diameter (mm)	3.17 ± 0.46	3.15 ± 0.47	0.722
Stent length (mm)	26.8 ± 12.3	27.3 ± 14.1	0.693
**Culprit coronary artery**			
Left anterior descending	207 (44.1%)	50 (40.7%)	0.458
Left circumflex	96 (20.5%)	22 (17.9%)	
Right	166 (35.4%)	51 (41.5%)	
CI-AKI	6 (1.3%)	38(30.9%)	<0.001
In hospital survival	458 (97.7%)	106 (86.2%)	<0.001

ACEI: angiotensin converting enzyme inhibitor; ARB: angiotensin receptor blocker; CI-AKI: contrast-induced acute kidney injury. Note: Values are presented as the mean ± SD, median (25-75 IQR) or number (%).

**Figure 2:** – ROC curve analysis of the glucose to lymphocyte ratio (GLR) for predicting contrast-induced acute kidney injury in ST-Elevation acute myocardial infarction patients; AUC: area under the curve; CI: confidence interval.

**Central Illustration:** Association between Glucose/lymphocytes Ratio and Contrast-Induced Acute Kidney Injury in Patients with Myocardial Infarction without Diabetes Mellitus

The univariate logistic regression analysis revealed that high-GLR, age, women gender, high BMI, arterial hypertension, active smoking, platelet count, hemoglobin, serum creatinine, LVEF, and contrast media volume were related to the increased incidence of CI-AKI. This finding was further substantiated by the results of the multivariate analysis. High-GLR still served as an independent predictor of CI-AKI in the study cohort after adjustment, with the odds ratio (OR) was 45.100 (p<0.001), together with creatinine (OR: 10.459, p=0.036) following adjustment for BMI, gender, age, comorbidities, LVEF, total amount of contrast and laboratory results ( Table 2 ).

**Table 2:** – Univariate and multivariate regression analyses for contrast-induced acute kidney injury (CI-AKI) in ST-Elevation myocardial infarction (STEMI)

Variable	Univariate analysis		Multivariate analysis	
	Odds ratio (95%CI)	p	Odds ratio (95%CI)	p
Age	1.095 (1.068-1.123)	<0.001	1.053 (0.977-1.135)	0.180
Women	3.387 (1.784-6.430)	<0.001	2.233 (0.318-15.685)	0.419
Body mass index	0.904 (0.818-0.998)	0.046	0.905 (0.738-1.109)	0.336
Arterial hypertension	3.597 (1.918-6.747)	<0.001	2.205 (0.437-11.131)	0.338
Hyperlipidemia	0.503 (0.208-1.216)	0.127		
Active smoking	0.219 (0.112-0.428)	<0.001	0.562 (0.67-4.6866)	0.594
White blood cell count	1.064 (0.992-1.142)	0.083		
GLR ≥ 4.16	34.498 (14.145-84.136)	<0.001	45.100 (7.312-278.174)	<0.001
Platelet count	0.994 (0.989-0.999)	0.019	0.992 (0.980-1.003)	0.159
Hs-CRP	1.008 (0.987-1.029)	0.469		
Serum creatinine	30.623 (9.659-97.090)	<0.001	10.459 (1.169-93.583)	0.036
Hemoglobin	0.967 (0.572-0.777)	<0.001	0.877 (0.570-1.350)	0.551
Systolic blood pressure	0.985 (0.971-1.001)	0.060		
Diastolic blood pressure	0.978 (0.955-1.001)	0.065		
Pulse rate	1.009 (0.989-1.030)	0.376		
LVEF	0.924 (0.893-0.956)	<0.001	0.921 (0.848-1.001)	0.053
Total ischemia time	1.002 (0.999-1.004)	0.170		
Contrast media volume	1.008 (1.004-1.012)	<0.001	1.007 (0.998-1.015)	0.116
Multivessel coronary lesions	0.611 (0.328-1.141)	0.122		
Stent diameter	0.714 (0.340-1.499)	0.374		
Stent length	1.005 (0.979-1.030)	0.722		

CI-AKI: contrast-induced acute kidney injury; STEMI: ST-Elevation myocardial infarction; CI: confidence interval; GLR: glucose to lymphocyte ratio; GLR: glucose to lymphocyte ratio; Hs-CRP: high sensitive C-reactive protein; LVEF: left ventricular ejection fraction.

## Discussion

The present analysis suggests GLR as an independent predictor of CI-AKI in non-diabetic STEMI patients managed with PPCI. This finding may have important diagnostic, therapeutic and prognostic implications in this group of patients.

The incidence of CI-AKI following PPCI largely depends on the baseline clinical and demographic features along with factors emerging during the intervention.^8^In agreement with previous studies, the overall CI-AKI incidence was 7.4% in our study. The mechanisms of CI-AKI primarily comprise direct renal tubular toxicity exerted by the contrast and a variety of renal hemodynamic alterations that lead to medullary hypoxia and inflammation,^18^and ultimately to the release of oxygen radicals, and vasoconstrictor and thrombogenic substances.^19^

CI-AKI has been a risk factor for unfavorable outcomes irrespective of the baseline renal function and a potential limitation for invasive cardiovascular interventions particularly in STEMI patients.^1 , 2 , 20^Mitigation of CI-AKI development in STEMI patients may significantly reduce postprocedural adverse events and thus improve clinical outcomes. Many researchers have demonstrated that certain hemogram-derived inflammatory biomarkers might play a crucial role in the prediction of CI-AKI risk in the setting of STEMI.^21 - 23^However, only a couple of studies have analyzed the combined impact of inflammatory factors and glucose in this context.

The GLR is a novel index that is reflective of both glucose metabolism and systemic inflammatory response and has been suggested as a promising risk marker in the setting of malignancy and intensive care unit.^9 - 14^However, few studies have explored the relevance of GLR in cardiovascular diseases. Importantly, this seems to be the first study to explore the relation between preinterventional GLR and CI-AKI in patients with STEMI (undergoing PPCI), suggesting that GLR may be an independent predictor of CI-AKI in these high-risk subjects. Therefore, this study has further increased the clinical usefulness of this index.

The absolute mechanisms regarding the independent relationship between GLR and CI-AKI is currently unknown. On the other hand, serum glucose is regarded as a metabolic marker potentially associated with the induction of persistent subtle inflammation.^24^Accumulating evidence has demonstrated that high glucose levels may trigger oxidative stress and associated chronic inflammation that might manifest as the expression of various proinflammatory mediators.^25 , 26^Under high-glucose circumstances, activated endothelial cells may release a variety of proinflammatory cytokines in response to paracrine and autocrine signals usually demonstrating a vicious cycle.^27^Moreover, high glycemia potentially induces glucose oxidation along with nonenzymatic protein glycation leading to disproportionately high production of free radicals.^28^However, antioxidant defense mechanisms may have the potential to prevent such pathological damage and emerging insulin resistance further triggered by the hyperglycemia-associated oxidative stress.^29^

On the other hand, as a crucial component of the systemic inflammatory response, decreased lymphocyte count, may be associated with poor cardiovascular prognosis in acute STEMI.^30^Several studies have suggested lymphocyte-based serum inflammatory biomarkers (including the neutrophil-to-lymphocyte ratio,^21^the systemic immune-inflammation index,^22^the prognostic nutritional index,^23^among others) as markers of CI-AKI development and unfavorable outcomes in patients in diverse populations. However, only a few reports have investigated the combined impact of proinflammatory factors and glucose as a metabolic factor. Taken together, GLR represents the synergistic impact of systemic inflammation and glucose, suggesting its unique clinical value. However, the clinical value of GLR in the prediction of CI-AKI should be further tested in STEMI patients as well as in other populations.

This study also has some limitations. First, this is a single-center analysis that might be inherently associated with selection biases even in the presence of large sample size. Second, we were not able to assess serial changes in the GLR value during the hospitalization period. Third, serum creatinine levels may be influenced by hemodynamic alterations even though there was no patient with cardiogenic shock in the study cohort. Finally, this is a retrospective analysis, potentially warranting prospective multicenter studies to confirm these findings.

## Conclusions

In the present study, we showed, for the first time, that admission GLR may act as an independent predictor of postprocedural CI-AKI in subjects with STEMI managed with PPCI. These results may impact clinical practice, including the possibility of using GLR in clinical protocols and reno-protective strategies, such as intravenous hydration and limited use of contrast media during the procedure. Further prospective studies are needed to better understand the mechanism of the relationship between high-GLR and CI-AKI.
